# Supplementary material for: Mechanistic Integration of Network Pharmacology and In Vivo Validation: TFRD Combat Osteoporosis via PI3K/AKT Pathway Activation
Source: Int J Mol Sci. 2025 Apr 12;26(8):3650. doi: 10.3390/ijms26083650 (PMC12027698; doi:10.3390/ijms26083650)
Supplement: Supplementary file 1 [file ijms-26-03650-s001.zip › ijms-3540015-supplementary.pdf]

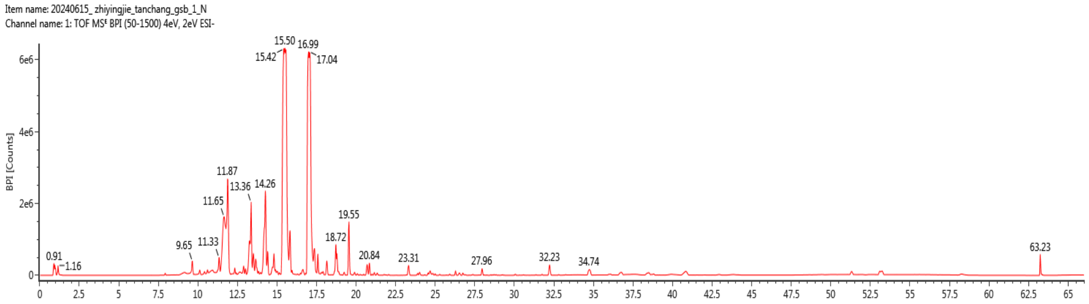

Figure S1.TIC diagram of the TFRD in negative ion mode.

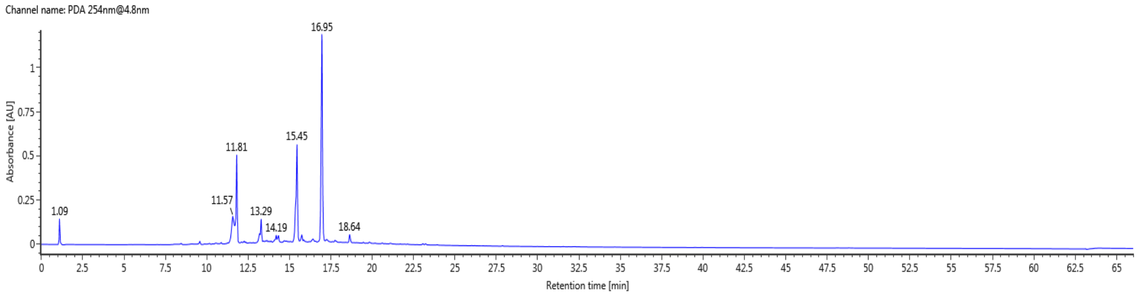

Figure S2. Ultraviolet image in 254nm mode

Table S1. The UPLC-MS/MS analysis results of TFRD

**PubChem CID**	**Component name**	**Formula**	**Neutral mass (Da)**	**Response**	**Adducts**
114627	Neoeriocitrin	$C_{27}H_{32}O_{15}$	596.17412	5629773	-H, +Cl
442428	Naringin	$C_{27}H_{32}O_{14}$	580.17921	4560328	-H, +Cl
5282152	Lonicerin	$C_{27}H_{30}O_{15}$	594.15847	1105193	-H, +Cl
90795788	Kaempferol 3-O-Rhamnoside-7-O-Glucoside	$C_{27}H_{30}O_{15}$	594.15847	926675	-H, +Cl
5281220	Aureusidin	$C_{15}H_{10}O_{6}$	286.04774	452746	-H
442431	Narirutin	$C_{27}H_{32}O_{14}$	580.17921	364009	-H
439246	Naringenin	$C_{15}H_{12}O_{5}$	272.06847	286911	-H
75227857	Kaempferol 3-O-beta-D-glucopyranoside-7-O-alpha-L-arabinofuranoside	$C_{26}H_{28}O_{15}$	580.14282	231662	-H
9064	Cianidanol	$C_{15}H_{14}O_{6}$	290.07904	184651	-H, +Cl
5280704	Cosmetin	$C_{21}H_{20}O_{10}$	432.10565	149192	-H
442615	Lucenin-2	$C_{27}H_{30}O_{16}$	610.15338	97283	-H, +CH3COO
440735	Eriodictyol	$C_{15}H_{12}O_{6}$	288.06339	86742	-H
147299	Procyanidin B4	$C_{30}H_{26}O_{12}$	578.14243	75680	-H
5280637	Cynaroside	$C_{21}H_{20}O_{11}$	448.10056	67321	-H
637542	p-Coumaric acid	$C_{9}H_{8}O_{3}$	164.04734	60567	+CH3COO, +e
444539	Cinnamic acid	$C_{9}H_{8}O_{2}$	148.05243	53997	+HCOO, +e
14825516	Linocaffein	$C_{15}H_{18}O_{9}$	342.09508	45427	-H

5282149	Trifolin	$C_{21}H_{20}O_{11}$	448.10056	43988	-H
44583907	5-Hydroxy-7-(beta-D-glucopyranosyloxy)chromone	$C_{15}H_{16}O_9$	340.07943	38784	-H
5281675	Orientin	$C_{21}H_{20}O_{11}$	448.10056	35292	-H
124017	Procyanidin B5	$C_{30}H_{26}O_{12}$	578.14243	35161	-H
5280445	Luteolin	$C_{15}H_{10}O_6$	286.04774	24348	-H, +e
5321164	Aureusidin-6-glucoside	$C_{21}H_{20}O_{11}$	448.10056	19164	-H
